# Micronuclei in Cord Blood Lymphocytes and Associations with Biomarkers of Exposure to Carcinogens and Hormonally Active Factors, Gene Polymorphisms, and Gene Expression: The NewGeneris Cohort

**DOI:** 10.1289/ehp.1206324

**Published:** 2013-11-19

**Authors:** Domenico Franco Merlo, Silvia Agramunt, Lívia Anna, Harrie Besselink, Maria Botsivali, Nigel J. Brady, Marcello Ceppi, Leda Chatzi, Bowang Chen, Ilse Decordier, Peter B. Farmer, Sarah Fleming, Vincenzo Fontana, Asta Försti, Eleni Fthenou, Fabio Gallo, Panagiotis Georgiadis, Hans Gmuender, Roger W. Godschalk, Berit Granum, Laura J. Hardie, Kari Hemminki, Kevin Hochstenbach, Lisbeth E. Knudsen, Manolis Kogevinas, Katalin Kovács, Soterios A. Kyrtopoulos, Martinus Løvik, Jeanette K Nielsen, Unni Cecilie Nygaard, Marie Pedersen, Per Rydberg, Bernadette Schoket, Dan Segerbäck, Rajinder Singh, Jordi Sunyer, Margareta Törnqvist, Henk van Loveren, Frederik J. van Schooten, Kim Vande Loock, Hans von Stedingk, John Wright, Jos C. Kleinjans, Micheline Kirsch-Volders, Joost H.M. van Delft, the NewGeneris Consortium

**Affiliations:** 1 Epidemiology, Biostatistics, and Clinical Trials, Istituto di Ricerca e Cura a Carattere Scientifico (IRCCS) Azienda Ospedaliera Universitaria (AOU) San Martino-Istituto Nazionale per la Ricerca sul Cancro (IST), Genoa, Italy; 2 Hospital del Mar Medical Research Institute, Barcelona, Spain; 3 Molecular and Environmental Epidemiology, National Institute of Environmental Health, Budapest, Hungary; 4 BioDetection Systems b.v., Amsterdam, the Netherlands; 5 National Hellenic Research Foundation, Institute of Biology, Medicinal Chemistry and Biotechnology, Athens, Greece; 6 Cancer Studies and Molecular Medicine, University of Leicester, Leicester, United Kingdom; 7 Department of Social Medicine, University of Crete, Heraklion, Crete, Greece; 8 Division of Molecular Genetic Epidemiology, German Cancer Research Center, Heidelberg, Germany; 9 Laboratory of Cell Genetics, Faculty of Science and Bio-engineering, Vrije Universiteit Brussel, Brussels, Belgium; 10 Division of Biostatistics, Leeds Institute for Genetics, Health and Therapeutics, University of Leeds, Leeds, United Kingdom; 11 Center for Primary Health Care Research, Lund University, Malmö, Sweden; 12 Genedata AG, Basel, Switzerland; 13 Department of Toxicology and Toxicogenomics, Maastricht University, Maastricht, the Netherlands; 14 Division of Environmental Medicine, Norwegian Institute of Public Health, Oslo, Norway; 15 Department of Public Health, University of Copenhagen, Copenhagen, Denmark; 16 Centre for Research in Environmental Epidemiology, Barcelona, Spain; 17 CIBER Epidemiología y Salud Pública, Barcelona, Spain; 18 National School of Public Health, Athens, Greece; 19 Environmental Chemistry Unit, Department of Materials and Environmental Chemistry, Stockholm University, Stockholm, Sweden; 20 Department of Biosciences and Nutrition, Karolinska Institute, Huddinge, Sweden; 21 Laboratory for Health Protection Research, National Institute of Public Health and the Environment, Bilthoven, the Netherlands

## Abstract

Background: Leukemia incidence has increased in recent decades among European children, suggesting that early-life environmental exposures play an important role in disease development.

Objectives: We investigated the hypothesis that childhood susceptibility may increase as a result of *in utero* exposure to carcinogens and hormonally acting factors. Using cord blood samples from the NewGeneris cohort, we examined associations between a range of biomarkers of carcinogen exposure and hormonally acting factors with micronuclei (MN) frequency as a proxy measure of cancer risk. Associations with gene expression and genotype were also explored.

Methods: DNA and protein adducts, gene expression profiles, circulating hormonally acting factors, and GWAS (genome-wide association study) data were investigated in relation to genomic damage measured by MN frequency in lymphocytes from 623 newborns enrolled between 2006 and 2010 across Europe.

Results: Malondialdehyde DNA adducts (M_1_dG) were associated with increased MN frequency in binucleated lymphocytes (MNBN), and exposure to androgenic, estrogenic, and dioxin-like compounds was associated with MN frequency in mononucleated lymphocytes (MNMONO), although no monotonic exposure–outcome relationship was observed. Lower frequencies of MNBN were associated with a 1-unit increase expression of *PDCD11, LATS2, TRIM13, CD28, SMC1A, IL7R,* and *NIPBL* genes. Gene expression was significantly higher in association with the highest versus lowest category of bulky and M_1_dG–DNA adducts for five and six genes, respectively. Gene expression levels were significantly lower for 11 genes in association with the highest versus lowest category of plasma AR CALUX® (chemically activated luciferase expression for androgens) (8 genes), ERα CALUX® (for estrogens) (2 genes), and DR CALUX® (for dioxins). Several SNPs (single-nucleotide polymorphisms) on chromosome 11 near *FOLH1* significantly modified associations between androgen activity and MNBN frequency. Polymorphisms in *EPHX1/2* and *CYP2E1* were associated with MNBN.

Conclusion: We measured *in utero* exposure to selected environmental carcinogens and circulating hormonally acting factors and detected associations with MN frequency in newborns circulating T lymphocytes. The results highlight mechanisms that may contribute to carcinogen-induced leukemia and require further research.

Citation: Merlo DF, Agramunt S, Anna L, Besselink H, Botsivali M, Brady NJ, Ceppi M, Chatzi L, Chen B, Decordier I, Farmer PB, Fleming S, Fontana V, Försti A, Fthenou E, Gallo F, Georgiadis P, Gmuender H, Godschalk RW, Granum B, Hardie LJ, Hemminki K, Hochstenbach K, Knudsen LE, Kogevinas M, Kovács K, Kyrtopoulos SA, Løvik M, Nielsen JK, Nygaard UC, Pedersen M, Rydberg P, Schoket B, Segerbäck D, Singh R, Sunyer J, Törnqvist M, van Loveren H, van Schooten FJ, Vande Loock K, von Stedingk H, Wright J, Kleinjans JC, Kirsch-Volders M, van Delft JHM, NewGeneris Consortium. 2014. Micronuclei in cord blood lymphocytes and associations with biomarkers of exposure to carcinogens and hormonally active factors, gene polymorphisms, and gene expression: The NewGeneris Cohort. Environ Health Perspect 122:193–200; http://dx.doi.org/10.1289/ehp.1206324

## Introduction

Cancer incidence among European children, specifically leukemia, has steadily increased over the last three decades (Kaatsch 2010). In view of the relatively short latent period for leukemia and its very early onset in childhood, it has been suggested that fetal exposure to environmental carcinogens may increase susceptibility to this cancer (Wild and Kleinjans 2003).

The European Union (EU)–funded project Newborns and Genotoxic exposure risks (NewGeneris) was designed to evaluate the hypothesis that maternal intake of dietary and other environmental carcinogens results in *in utero* exposure and early biological effects in the unborn child, possibly leading to increased risk of cancer in later childhood (Merlo et al. 2009). The primary aim of the present study was to investigate the relationship between biomarkers of exposure to carcinogenic compounds and micronuclei (MN) frequency in umbilical cord blood lymphocytes from the NewGeneris mother–child birth cohort. The secondary aim was to ascertain whether individual genotypes modify these relationships.

An array of exposure biomarkers quantifying a range of potentially carcinogenic exposures, many of dietary origin, were measured in fetal cord blood samples. DNA and hemoglobin (Hb) adducts reflect biologically effective doses of exposure to genotoxic agents (Phillips 2008). The DNA adducts selected for study were 3-(2´-deoxy-β-d-erythro-pentofuranosyl)-pyrimido[1,2-α]purine-10(3H)-one (M_1_dG), O^6^-methyldeoxyguanosine (O^6^-MedG), and bulky DNA adducts. Hb adducts from acrylamide (AA), glycidamide (GA), and ethylene oxide (EtO) [for which data in this cohort have been published before (Pedersen et al. 2012)] were also evaluated, and three versions of the chemically activated luciferase gene expression (CALUX®) bioassay were used to assess exposure to androgenic (AR), estrogenic (ERα), and dioxin-like (DR) compounds.

Facilitated by the development of microarray technologies, gene expression–based biomarkers have been developed and applied for human biomonitoring purposes (McHale et al. 2011; Rager et al. 2011; Ren et al. 2011; van Leeuwen et al. 2008). Gene expression profiling has the potential to identify new biomarkers of exposure that may simultaneously reflect the earliest biological events in disease pathogenesis. Here, we evaluated the expression of 36 genes that were associated with biomarkers of carcinogen exposure by quantitative real-time polymerase chain reaction (qRT-PCR) (Hochstenbach et al. 2012).

MN frequency was assessed as the primary outcome. MN are a potential biomarker of cancer risk, because increased micronucleated binucleated (MNBN) frequencies in T lymphocytes have been shown to be associated with cancer risk in adults (Bonassi et al. 2007). MN are small extranuclear bodies arising in dividing cells that are caused by chromosomal breakage and/or whole chromosome loss (Fenech 2007; Kirsch-Volders et al. 2011). MNBN provide a measure of the lesions that have recently occurred *in vivo*, whereas micronucleated mononucleated lymphocytes (MNMONO) give an estimation of the genome damage accumulated over a long period in stem cells and circulating lymphocytes (Kirsch-Volders and Fenech 2001).

Furthermore, we performed a genome-wide association study (GWAS) to investigate whether associations between exposure biomarkers and MN are modified by genetic variation.

## Materials and Methods

*Study population and sample collection*. Pregnant women (*n* = 1,200) were enrolled between 2006 and 2010 in Heraklion, Crete, Greece; Barcelona and Sabadell, Spain; Bradford, England; Copenhagen, Denmark; and Oslo and Akerhus, Norway (Pedersen et al. 2012). The participation of mothers in the study was based on previously described eligibility criteria (Pedersen et al. 2012). Study protocols were approved by local ethics committees, and informed consent was obtained from all participating mothers before sample collection.

Detailed information on personal characteristics, including demographic, health, and lifestyle factors, was obtained using extensive questionnaires completed by mothers before or around the time of delivery. Information on dietary habits during pregnancy was obtained from country-specific food-frequency questionnaires (FFQs). Information on birth weight, gestational age, sex, and type of delivery was obtained from maternity records. Gestational age (completed weeks) was computed based on last menstrual period or ultrasound-based estimated date of conception.

Blood samples were collected from 1,151 mother–infant dyads following a common protocol as described previously (Merlo et al. 2009). Umbilical cord blood samples were collected immediately after birth from the cord vein of newborns and locally processed. Samples were kept at –20°C or –80°C until shipment on dry ice to the study laboratories.

*Biomarkers of exposure and early biological effect*. DR CALUX® bioassay. Dioxin-like activity, expressed as aryl hydrocarbon receptor (AhR)–mediated activation of the extractable lipid fraction from plasma, was determined through the DR CALUX bioassay developed by BioDetection System (Murk et al. 1996). Blood was collected in heparinized tubes and plasma was isolated by centrifugation on the day of collection and frozen at –20^o^C. One to three milliliters of cord blood plasma was used for extraction of lipophilic compounds. The procedure for the DR CALUX® bioassay has been described in detail previously (Behnisch 2005). Additional information is provided in Supplemental Material (p. 3).

ERα and AR CALUX® bioassays. Estrogenic and androgenic activity in cord-blood plasma was determined using the ERα and AR CALUX® Bioassays. The ERα and AR CALUX® bioassays comprise human bone cell lines (U2OS), stably incorporating the firefly luciferase gene coupled to responsive elements (REs) as a reporter gene for the presence of (xeno-) estrogens (ERα CALUX®) and androgens (AR CALUX®) (Sonneveld et al. 2005). Additional information is provided in Supplemental Material (p. 4).

Hb adducts. Erythrocytes were isolated by centrifugation on the day of collection and stored at –20°C. AA–, GA–, and EtO–Hb adducts were simultaneously determined by the adduct FIRE procedure using liquid chromatography tandem mass spectrometry with performance and validation standards as described in detail elsewhere (von Stedingk et al. 2010, 2011). In total, Hb adduct levels were measured in 1,151 cord blood samples.

*DNA adducts*. DNA was isolated with the Qiagen Midi Kit (no. 13343; Qiagen, Hilden, Germany) with some modifications of the manufacturer’s protocol, as reported previously (Kovács et al. 2011). Additional details are provided in Supplemental Material (pp. 4–8).

Immunoslot blot analysis of M_1_dG. M_1_dG was determined by an immunoslot blot method, using a murine M_1_dG monoclonal primary antibody (D10A1), provided by L. Marnett (Vanderbilt University, TN, USA), as described previously (Singh et al. 2001).

Immunochemical assays for analyses of O^6^-MedG and PAH–DNA adducts. These analyses were carried out using ultrasensitive sandwich chemiluminescence immunoassays as previously described for O^6^-MedG (Georgiadis et al. 2011) and PAH (polycyclic aromatic hydrocarbons)–DNA adducts (Georgiadis et al. 2012).

Postlabeling analysis of bulky DNA adducts. Bulky DNA adducts were detected with the nuclease P1 modification of the ^32^P-postlabeling procedure as detailed elsewhere (Kovács et al. 2011). Interlaboratory differences in levels were adjusted for, as described in Supplemental Material (p. 8).

*Cytokinesis block micronucleus assay*. The *in vitro* cytokinesis blocked MN assay was carried out according to the standardized protocol developed for semiautomated image analysis (Decordier et al. 2009) and adapted for umbilical blood (Vande Loock et al. 2011). MN were scored in both MNBN and MNMONO T lymphocytes (Kirsch-Volders and Fenech 2001). To harmonize slide preparation, the cohort cytologists were trained by I.D., K.V.L., and M.K.-V. (Vrije Universiteit Brussel; VUB). Slides were sent to VUB, where staining and MN analysis occurred. Quality control after staining included visual selection of slides with good quality, using a light microscope and based on a good spreading, swelling, and amount of cells. The automated scoring procedure followed by visual validation of selected micronucleated cells was carried out by the same researcher, using the PathFinder™ platform installed by IMSTAR S.A. (Paris, France) at the VUB laboratory; this consisted of a PathFinder™ CELLSCAN™ capture station and two PathFinder™ MN analysis workstations. Reproducibility of the automated image analysis combined with the visual validation step was investigated by assessing the intercapturing variability (Decordier et al. 2009, 2011). At the end of the processing step, cells containing detected MN are presented one by one on the screen and confirmed or rejected by the scorer, according to the Human MicroNucleus project scoring criteria (Fenech et al. 2003). According to guideline T487 of the Organisation for Economic Co-operation and Development (OECD), only subjects with at least 1,000 BN T lymphocytes counted were considered for statistical analysis (OECD 2010).

*Gene expression analysis*. To preserve RNA for gene expression analysis, 0.4 mL of heparin-anticoagulated whole blood was mixed with 1.2 mL RNAlater (Ambion/Applied Biosystems, Nieuwerkerk aan den Ijssel, the Netherlands) as soon as possible after blood collection. Samples were kept at –80°C until shipment on dry ice to the research laboratory at Maastricht University. Total RNA was isolated using the RiboPure-Blood system (Ambion) according to the manufacturer’s instructions. RNA integrity was verified by gel electrophoresis (2100 BioAnalyzer; Agilent Technologies, Amstelveen, the Netherlands).

Fluidigm’s BioMark™ Dynamic Array (Fluidigm, Amsterdam, the Netherlands) technology was used for gene expression analyses by qRT-PCR, which was conducted by ServiceXS (Leiden, the Netherlands). Thirty-six genes were selected from a whole genome gene–environment interaction study on neonates (*n* = 84) from the Norwegian cohort (Hochstenbach et al. 2012). Selection was primarily based on correlations (*r* ≥ 0.75, ≤ –0.75, *p* < 0.05) of gene expression with toxic dietary exposures (i.e., genotoxic or immunotoxic) estimated based on FFQs, CALUX assay–based evidence of exposure to estrogenic-, androgenic-, and dioxin-like compounds, Hb adduct levels, and MN frequencies. Only mechanistically relevant genes were selected, based on gene ontologies such as DNA repair, cell cycle, apoptosis, and cell proliferation. For each of the correlations, mechanistically relevant genes were selected, resulting in 36 unique genes. Five reference genes were selected, based on low variance across all individuals. TaqMan gene expression assays (Applied Biosystems) were used (see Supplemental Material, Table S1), and qRT-PCR was conducted according to the manufacturer’s protocol. Each sample was analyzed in duplicate, and an average C_t_ (threshold cycle) value obtained. On all RT-PCR plates, a reference sample at various dilutions was included for quality control assessment of interplate reproducibility. The raw C_t_ value upper cut-off was set to 26; genes exceeding this value were classified as unexpressed. For normalization, the average C_t_ of the five reference genes was subtracted from the C_t_ value of each gene.

*Genome-wide association studies and candidate genes analyses*. We conducted a genome-wide scan of approximately 300,000 tagging single nucleotide polymorphisms (SNPs) using the Illumina HumanCytoSNP-12 v1 (Illumina Inc., Hayward, CA, USA) according to the manufacturer’s protocols. Genotype calling was done using Illumina GenomeStudio 2010. Genomic DNA was isolated from 900 cord blood samples and was used to genotype each child. Quality control was performed on a per-sample and per-SNP basis. We excluded 33 duplicates, 23 samples with a genotype call rate < 98.5%, and 14 twins, leaving 830 genotyped samples available for analysis. We used a general genetic model retaining the three distinct genotypes and without making any assumption about the direction of the SNP’s association in the heterozygote compared with the two homozygote classes. According to nonmutually exclusive SNP-based quality checks, 6,801 SNPs were excluded because of Hardy–Weinberg equilibrium violation (*p* < 10^–6^), 35,429 because they had a minor allele frequency (MAF < 1%), and 7,338 because missing genotype was > 10%, resulting in 258,246 of 298,199 SNPs left for statistical analyses. A total of 435 newborns had both SNPs and MN results available, and they were used in GWAS statistical analyses.

In addition, SNPs present in metabolism and DNA repair genes were selected *a priori* by the consortium as candidate genes based on the available knowledge on functionalities with respect to bioactivation (*CYP1A1*, *CYP2E1*, *CYP2D6*, *EPHX1*, and *EPHX2*) and detoxification (*GSTM1*) of DNA adduct–forming metabolites, base excision repair of oxidative adducts (*OGG1*), nucleotide excision repair of bulky adducts (*XRCC1*, *ERCC2/XPD*, *XPA*, and *XRCC3*), repair of alkylated adducts (*MGMT*, *ALKB*, and *MPG*) and of thymine adducts (*TDG*), and with respect to folate metabolism, which is known to interfere with micronucleus formation (*MTHFR, MTR*, and *MTRR*).

*Statistical analyses*. Separate negative binomial fixed effects multivariable regression models were used to estimate the associations of MN frequencies per 1,000 MNBN or MNMONO T lymphocytes (as dependent variables) with AA–Hb, GA–Hb, and EtO–Hb adducts (picomoles per gram Hb, categorized into quintiles); M_1_dG–, PAH–, bulky, and O^6^-MedG–DNA adducts (per 10^8^ nucleotides, categorized into quartiles); and plasma levels of AR CALUX® (nanograms DHT AEQ/milliliters plasma), ERα CALUX® [nanograms ERα estradiol equivalents (EEQ)/milliliters plasma], and DR CALUX® (picograms TEQ/milliliters plasma) (categorized into quartiles); gene expression (normalized C_t_ value, continuous), GWAS data (i.e., all SNPs and *a priori* selected candidate genes), and the interactions between biomarkers and SNPs.

Cohort (country), maternal age (continuous), gestational age (continuous), prepregnancy maternal body mass index (continuous), maternal smoking during pregnancy (any or none), environmental tobacco smoke (ETS) exposure during pregnancy (any or none), maternal ethnicity (Caucasian, others), and newborn sex and birth weight (continuous) were selected as potential confounders *a priori* and included in all models. Observations with missing covariates were excluded from the statistical analysis. We report the relative difference in the frequency of MN for each category of exposure relative to the lowest exposure category and the associations between 1-unit increases in gene expression and MN frequency as the mean ratio (MR) and its 95% CI. The likelihood ratio test was used as a global test of statistical significance over all categories of each exposure biomarker, SNPs allele variants, and the interactions between exposure biomarkers with gene expression and with SNPs.

We estimated associations between the expression of each of the 36 genes evaluated and categorical exposure biomarkers using separate multivariable linear regression models adjusted for the covariates listed above. The *F*-test was used as a global test of statistical significance over all categories of each exposure biomarker. For each exposure biomarker we report the differences in gene expression associated with the highest versus lowest category of exposure biomarkers.

For gene expression and GWAS analyses, we adjusted the estimated *p*-values to account for multiple comparisons using standard methods (Benjamini and Hochberg 1995; Hochberg 1988; Holm 1979). This criterion was used to identify SNPs associated with MN as main predictors or as effect modifiers of the exposure biomarkers–MN and gene expression–MN associations. No adjustment was made for *p*-values estimated from the analyses of *a priori*–selected candidate genes. *p*-Values < 0.05 were considered statistically significant. All associations were examined in newborns with MN assay data available (*n* = 623) and with exposure biomarkers, gene expression, and GWAS data available. Sample sizes for individual association analyses varied as indicated in the results.

Manhattan plots for *p*-values along chromosome and position were made by the genetic analysis package (gap) for CRAN R 2.11. Statistical analyses were carried out using Stata S.E. version 10.0 (StataCorp, College Station, TX, USA), R (http://cran.r-project.org), and Genedata Expressionist 7.0 (Genedata AG, Basel, Switzerland).

## Results

Levels of biomarkers of exposure (i.e., Hb and DNA adducts, and AR, ERα, and DR CALUX® activity) detected in newborns are reported in Table 1. The number of observations for each biomarker varied reflecting the variable amount of biological specimens collected from cord blood and the assays prioritization adopted (i.e., Hb adducts, DNA adducts, and CALUX® activity). The largest number of observations was available for AA–Hb adducts (*n* = 1,151) and the smallest for DR CALUX® (*n* = 725). For all biomarkers large variations were present (e.g., AA–Hb adducts: median = 14.4 pmol/g Hb; range, 4.4–124.8; M_1_dG-DNA adducts: median = 9.9/10^8^ nucleotides; range, 0.5–324.7).

**Table 1:** Biomarkers of exposure measured in cord blood: descriptive statistics.

Biomarker of exposure (unit)	*n*	Mean ± SD	Median	IQR	Min, Max
Acrylamide–Hb adducts (pmol/g Hb)	1,151	19.7 ± 16.5	14.4	10.8, 21.6	4.4, 124.8
Glycidamide–Hb adducts (pmol/g Hb)	1,150	13.7 ± 10.4	10.8	7.9, 15.7	2.0, 103.2
Ethylene oxide–Hb adducts (pmol/g Hb)	1,123	13.3 ± 13.8	9.4	6.6, 14.5	0.5, 174.2
M_1_dG (per 10^8 ^nucleotides)	892	53.1 ± 52.5	34.2	12.2, 82.7	0.5, 324.7
PAH–DNA adducts (per 10^8^ nucleotides)	797	1.89 ± 2.09	1.39	0.58, 2.55	0.2, 28.3
Bulky DNA adducts (per 10^8^ nucleotides)	635	11.8 ± 11.2	8.4	4.8, 15.3	0.6, 116.6
O^6^-MedG (per 10^8^ nucleotides)	865	0.470 ± 0.501	0.287	0.075, 0.665	0.015, 3.033
AR CALUX^®^ (ng DHT AEQ/mL plasma)	765	0.108 ± 0.098	0.099	0.059, 0.140	0.015, 2.142
ERα CALUX^®^ (ng 17-β-estradiol EEQ/mL plasma)	765	22.1 ± 19.4	18.2	9.5, 28.5	0.01, 182.7
DR CALUX^®^ (pg TEQ/mL plasma)	725	0.165 ± 0.137	0.130	0.055, 0.230	0.055, 1.043
Abbreviations: IQR, interquartile range; Max, maximum; Min, minimum.					

Descriptive statistics for MNBN and MNMONO T lymphocytes are shown in Table 2 by cohort and by sociodemographic, reproductive, and lifestyle factors. Again large interindividual variations were observed within and between cohorts, with the highest level of MN observed in Greece (MNBN mean = 1.79 ± 1.50 per 1,000 binucleated T lymphocytes) and the lowest in the United Kingdom (MNBN mean = 0.55 ± 0.74).

**Table 2:** MNBN and MNMONO T lymphocytes measured in cord blood (*n* = 623) by sociodemographic, reproductive, and lifestyle factors: descriptive statistics (mean ± SD).

Covariates	*n* (%)	MNBN^*a*^	MNMONO^*a*^
Country
United Kingdom	143 (22.9)	0.55 ± 0.74	0.04 ± 0.14
Greece	232 (37.2)	1.79 ± 1.50	0.62 ± 0.71
Denmark	142 (22.7)	0.70 ± 0.58	0.17 ± 0.58
Spain	70 (11.2)	1.00 ± 1.00	0.20 ± 0.45
Norway	36 (5.77)	1.16 ± 0.92	0.11 ± 0.42
Maternal age (years)
≤ 27	170 (27.2)	1.31 ± 1.26	0.34 ± 0.59
28–30	131 (21.0)	1.11 ± 1.36	0.27 ± 0.53
31–32	96 (15.4)	1.11 ± 1.09	0.34 ± 0.61
33–35	109 (17.4)	1.17 ± 1.34	0.37 ± 0.73
≥ 36	112 (17.9)	0.85 ± 0.84	0.21 ± 0.55
Unknown	5 (0.80)	1.15 ± 1.43	0.20 ± 0.38
Prepregnancy BMI (kg/m^3^)
≤ 18.5 (underweight)	27 (4.33)	1.44 ± 1.19	0.39 ± 0.51
18.6–25.0 (normal)	316 (50.7)	1.20 ± 1.19	0.33 ± 0.65
25.1–30.0 (overweight)	112 (17.9)	1.27±1.36	0.25 ± 0.44
> 30 (obese)	78 (12.5)	1.07 ± 1.28	0.47 ± 0.78
Unknown	90 (14.4)	0.68 ± 0.92	0.13 ± 0.32
Birth weight (g)
Normal (≥ 2,500)	601 (96.4)	1.13 ± 1.22	0.31 ± 0.60
Low (< 2,500)	17 (2.72)	1.24 ± 0.88	0.37 ± 0.49
Unknown	5 (0.80)	1.32 ± 1.83	0.19 ± 0.38
Child sex
Male	315 (50.5)	1.10 ± 1.22	0.32 ± 0.63
Female	306 (49.1)	1.16 ± 1.21	0.30 ± 0.57
Unknown	2 (0.32)	0.37 ± 0.07	0.00 ± 0.00
Maternal ethnicity
Caucasian	522 (83.7)	1.21 ± 1.26	0.35 ± 0.64
Others	93 (14.9)	0.72 ± 0.86	0.08 ± 0.28
Unknown	8 (1.28)	0.44 ± 0.45	0.00 ± 0.00
Gestational age (weeks)
< 37	29 (4.65)	1.32 ± 1.62	0.30 ± 0.38
≥ 37	592 (95.0)	1.12 ± 1.19	0.31 ± 0.61
Unknown	2 (0.32)	1.22 ± 1.13	0.00 ± 0.00
Delivery
Vaginal	287 (46.0)	1.24 ± 1.26	0.35 ± 0.67
Caesarean	334 (53.6)	1.04 ± 1.17	0.27 ± 0.54
Unknown	2 (0.32)	0.48 ± 0.08	0.00 ± 0.00
Maternal smoking during pregnancy
None	470 (75.4)	1.06 ± 1.16	0.27 ± 0.58
Any	136 (21.8)	1.39 ± 1.37	0.47 ± 0.66
Unknown	17 (2.72)	0.89 ± 1.07	0.09 ± 0.25
ETS exposure during pregnancy
None	285 (45.7)	0.91 ± 0.91	0.18 ± 0.48
Any	231 (37.0)	1.26 ± 1.43	0.39 ± 0.68
Unknown	107 (17.1)	1.45 ± 1.32	0.46 ± 0.65
Abbreviations: BMI, body mass index; ETS, environmental tobacco smoke. ^***a***^Number per 1,000 binucleated or mononucleated T lymphocytes.			

None of the global tests of associations across all categories of exposure were statistically significant, and there was no evidence of monotonic dose–response trends with increasing levels of exposure for associations of AA–, GA–, or EtO–Hb adducts (quintiles); PAH–, bulky–, or O^6^-MG–DNA adducts (quartiles); or DR CALUX® plasma levels (quartiles) and frequencies of MNBN and MNMONO T lymphocytes (Table 3). A significant overall association was found between M_1_dG levels and the frequency of MNBN lymphocytes, although associations relative to the lowest quartile of M1dG were positive for the second and third quartiles and negative for the highest quartile. ERα CALUX® plasma levels were significantly associated with the frequency of MNBN and MNMONO lymphocytes and AR CALUX® with the frequency of MNMONO lymphocytes. No monotonic exposure–outcome association was observed between ERα CALUX® or AR CALUX® and MN. For ERα CALUX® a significant negative association with MNBN was detected for the second quartile, followed by a weak nonsignificant positive association with the third and fourth quartiles while the associations with MNMONO were negative for the second and fourth quartiles. The strongest associations were detected for AR CALUX® and MNMONO T lymphocytes and were positive for the second and third quartiles and negative for the fourth quartile.

**Table 3:** Relative difference in the frequency of MNBN and MNMONO T lymphocytes [mean ratio (MRa)] by category of exposure biomarkers estimated by negative binomial regression.

Exposure biomarker (unit) and category	*n* (%)	MNBN^*b*^MR (95% CI)	*p*‑Value^*c*^	MNMONO^*b*^MR (95% CI)	*p*‑Value^*c*^
Acrylamide–Hb adducts (pmol/g Hb)			0.468		0.873
≤ 10.2	95 (20.3)	1		1
10.3–13.9	93 (19.9)	1.06 (0.83, 1.35)		0.79 (0.48, 1.30)
14.0–18.4	94 (20.1)	1.00 (0.78, 1.28)		0.98 (0.62, 1.55)
18.5–27.8	93 (19.9)	0.97 (0.74, 1.27)		0.87 (0.53, 1.43)
> 27.8	92 (19.7)	1.24 (0.92, 1.68)		0.83 (0.46, 1.48)
Total	467 (100)
Glycidamide–Hb adducts (pmol/g Hb)			0.079		0.429
≤ 7.4	91 (19.4)	1		1
7.5–9.5	97 (20.7)	1.05 (0.83, 1.35)		1.17 (0.71, 1.94)
9.6–13.1	93 (19.9)	1.09 (0.85, 1.40)		1.10 (0.67, 1.82)
13.2–18.5	95 (20.3)	0.77 (0.58, 1.02)		0.74 (0.42, 1.31)
> 18.5	91 (19.4)	1.03 (0.76, 1.40)		0.79 (0.44, 1.41)
Total	467 (100)
Ethylene oxide–Hb adducts (pmol/g Hb)			0.336		0.676
≤ 6.3	89 (19.4)	1		1
6.4–8.5	94 (20.5)	1.22 (0.94, 1.58)		0.95 (0.55, 1.65)
8.6–11.5	93 (20.3)	1.22 (0.94, 1.59)		1.12 (0.64, 1.96)
11.6–16.8	91 (19.9)	1.08 (0.83, 1.41)		1.19 (0.70, 2.03)
> 16.8	90 (19.6)	1.01 (0.76, 1.33)		1.39 (0.81, 2.38)
Total	457 (100)
PAH–DNA adducts (per 10^8^ nucleotides)			0.133		0.724
≤ 6.3	85 (24.5)	1		1
6.4–8.5	87 (25.1)	0.92 (0.71, 1.18)		0.97 (0.62, 1.53)
8.6–11.5	87 (25.1)	0.74 (0.56, 0.97)		1.20 (0.71, 2.01)
11.6–16.8	87 (25.1)	0.98 (0.76, 1.28)		0.89 (0.57, 1.41)
Total	346 (100)
Bulky DNA adducts (per 10^8^ nucleotides)			0.096		0.757
≤ 4.60	77 (25.4)	1		1
4.70–7.90	73 (24.0)	1.20 (0.91, 1.59)		0.75 (0.37, 1.54)
8.00–14.78	77 (25.4)	0.90 (0.68, 1.20)		0.78 (0.41, 1.49)
> 14.78	76 (25.0)	0.85 (0.64, 1.14)		0.72 (0.40, 1.30)
Total	303 (100)
M_1_dG (per 10^8^ nucleotides)			0.024		0.761
≤ 12.02	94 (24.6)	1		1
12.03–37.78	95 (24.9)	1.16 (0.91, 1.49)		0.99 (0.66, 1.49)
37.79–83.75	96 (25.1)	1.29 (0.99, 1.67)		1.18 (0.74, 1.88)
> 83.75	96 (25.1)	0.89 (0.69, 1.15)		0.90 (0.58, 1.41)
Total	381 (100)
O^6^-MedG (per 10^8^ nucleotides)			0.539		0.111
≤ 0.076	133 (35.5)	1		1
0.077–0.389	79 (21.1)	0.99 (0.80, 1.23)		0.88 (0.60, 1.29)
0.390–0.810	81 (21.6)	1.16 (0.93, 1.45)		1.08 (0.73, 1.61)
> 0.810	81 (21.6)	1.06 (0.85, 1.34)		1.50 (1.00, 2.24)
Total	374 (100)
AR CALUX^®^ (ng DHT AEQ/mL plasma)			0.103		0.002
≤ 0.086	65 (25.0)	1		1
0.087–0.118	63 (24.3)	1.46 (1.07, 2.00)		2.17 (0.99, 4.74)
0.119–0.151	67 (25.7)	1.37 (0.99, 1.92)		2.63 (1.19, 5.78)
> 0.151	65 (25.0)	1.38 (0.97, 1.96)		0.78 (0.28, 2.17)
Total	260 (100)
ERα CALUX^®^ (ng 17-β-estradiol EEQ/mL plasma)			0.018		0.014
≤ 11.61	64 (24.8)	1		1
11.62–18.41	64 (24.8)	0.66 (0.46, 0.95)		0.54 (0.25, 1.20)
18.42–30.00	65 (25.1)	1.04 (0.77, 1.42)		1.41 (0.74, 2.70)
> 30.00	65 (25.1)	1.09 (0.78, 1.51)		0.57 (0.22, 1.51)
Total	258 (100)
DR CALUX^®^ (pg TEQ/mL plasma)			0.182		0.355
≤ 0.055	73 (29.6)	1		1
0.056–0.180	49 (19.9)	0.79 (0.55, 1.12)		1.50 (0.81, 2.77)
0.181–0.280	63 (25.6)	1.14 (0.85, 1.54)		1.10 (0.57, 2.11)
> 0.281	61 (24.7)	1.12 (0.78, 1.61)		1.74 (0.83, 3.65)
Total	246 (100)
^***a***^Mean ratio adjusted for country, maternal age, prepregnancy BMI, birth weight, sex, maternal ethnicity, gestational age, delivery, maternal smoking, and ETS. ^***b***^Number per 1,000 binucleated or mononucleated T lymphocytes. ^***c***^Log likelihood ratio test.					

**Figure 1:**
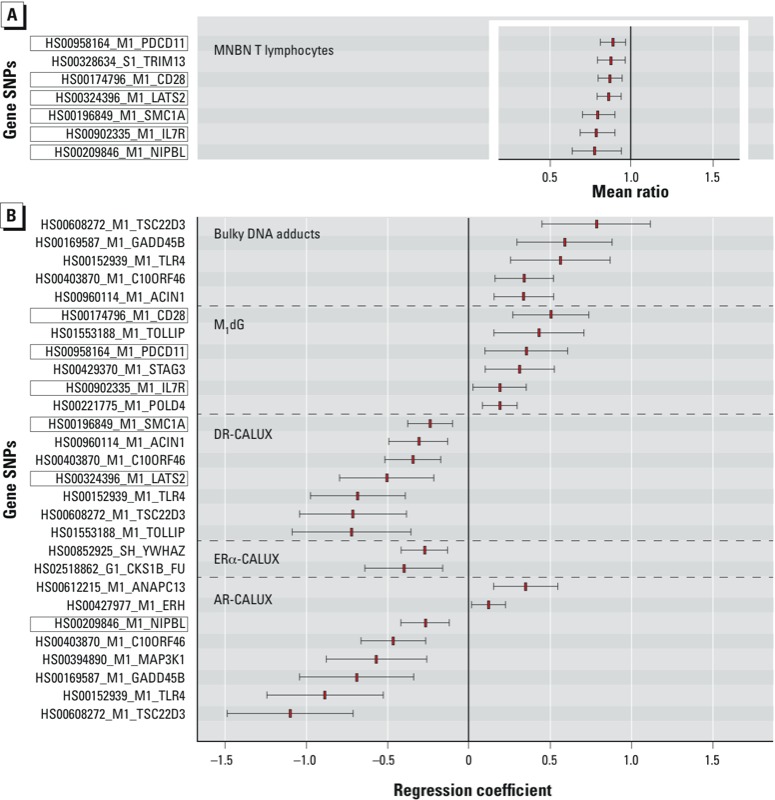
Associations between gene expression and MNBN T-lymphocyte frequency (*A*) and between exposure biomarkers and gene expression (*B*) adjusted for country, maternal age, prepregnancy BMI, birth weight, sex, maternal ethnicity, gestational age, delivery, maternal smoking, and ETS. Associations shown are those with multiple comparisons–adjusted *p*-values < 0.05. (*A*) Mean ratios (MR) are for associations between 1-unit increases in gene expression and MNBN based on 350 observations with complete data. (*B*) Differences in gene expression associated with the highest versus lowest category of exposure biomarkers are based on observations with complete data (bulky DNA adducts *n *= 398, M_1_dG DNA adducts *n *= 533, AR CALUX^®^ *n *= 457; DR CALUX^®^ *n *= 477; ER CALUX^®^ *n *= 457). DR CALUX^®^ categories were based on the following centiles: ≤ 0.13; 0.131–0.23; > 0.23 (pg TEQ/mL plasma) because 46% of the observations were tied (i.e., below the limit of detection of the assay). Boxed genes are significant predictors of MNBN and are significantly predicted by at least one exposure biomarker. Red rectangles are mean ratio point estimates of the associations between 1-unit increases in gene expression and MNBN (*A*), and regression coefficient point estimates of the differences in gene expression associated with the highest versus lowest category of exposure biomarkers (*B*). Whiskers are 95% CIs.

One-unit increases in the expression of 7 of the 36 genes evaluated (*PDCD11*, *LATS2*, *TRIM13*, *CD28*, *SMC1A*, *IL7R*, and *NIPBL*) were associated with significantly lower MNBN frequencies, with MR ranging from 0.81 (95% CI: 0.88, 0.96) for PDCD11 to 0.64 (95% CI: 0.77, 0.97) for NIPBL (Figure 1A). The frequency of MNMONO was not significantly associated with expression of any of the genes tested (data not shown).

In models with gene expression levels as the dependent variable, expression was significantly higher in association with the highest versus lowest category of bulky DNA adducts and of M_1_dG levels for five and six genes, respectively (Figure 1B). Conversely, expression levels were significantly lower for a total of 11 genes in association with the highest versus lowest category of plasma AR CALUX® (eight genes), ERα CALUX® (two genes), and DR CALUX® (seven genes) (Figure 1B). Associations with lower levels of exposure are not reported. Six of the seven genes whose expression was associated with significantly lower MNBN frequency (i.e., all except TRIM13; Figure 1A) were significantly associated with the highest versus lowest category of at least one exposure biomarker (M_1_dG, DR CALUX®, ERα CALUX®, or AR CALUX®; Figure 1B).

GWAS was carried out on 435 newborns with data available for both SNPs and micronuclei. Confounding by population stratification was assessed (see Supplemental Material, Figures S1 and S2) and confirmed that genotype variations occurred between population subgroups (i.e., maternal ethnicity and newborns’ country of birth), justifying the need for adjustment in statistical analyses. None of the GWAS SNPs were significant predictors of MNBN frequencies (see Supplemental Material, Figure S3). Investigation of the exposure biomarkers–SNPs interactions on the occurrence of MNBN revealed a cluster of significant SNPs (on chromosome 11) for AR CALUX® modeled as a continuous variable (see Supplemental Material, Figure S4). The four SNPs acting as effect modifiers of the relationship between AR CALUX® and the frequency of MNBN lymphocytes are given in Supplemental Material, Table S2. The association of these SNPs were reported per unit increase of plasma AR CALUX® and varied according to the allele variants. For each of the SNPs shown, there was a significant positive association between a 1-unit increase in plasma AR CALUX® and MNBN frequency among participants with one homozygous genotype, and a significant negative association with the alternate homozygous genotype (e.g., for rs7131537, MR = 2.54; 95% CI: 1.69, 3.75 for CC and MR = 0.36; 95% CI: 0.21, 0.60 for AA, with a null association among AC heterozygotes compared with an overall estimated association MR = 1.14; 95% CI: 0.88, 1.47; data not shown).

Furthermore, 89 SNPs from the 18 *a priori* selected candidate genes were investigated for association with MN frequencies. SNPs in *EPHX1*, *EPHX2*, and *CYP2E1* were significantly associated (unadjusted overall *p*-value < 0.05) with the frequency of MNBN lymphocytes (Table 4). None of the candidate-gene SNPs were significantly associated with the frequency of MNMONO lymphocytes (data not shown).

**Table 4:** Relationships between the available SNPs for the *a priori*–selected *EPHX* and *CYP2E1* genes and frequency of MNBN T lymphocytes in newborns.

SNP	Gene	*n*	MNBN^*a*^(mean ± SE)	MR (95 CI%)^*b*^	*p*‑Value^*c*^
Rs1051741	*EPHX1*	424			0.011
GG		330	1.20 ± 0.07	1
AG		85	1.12 ± 0.11	1.04 (0.85, 1.27)
AA		9	0.32 ± 0.15	0.35 (0.17, 0.71)
Rs4149244	*EPHX2*	434			0.032
GG		377	1.17 ± 0.06	1
AG		50	1.04 ± 0.16	0.85 (0.66, 1.08)
AA		7	1.86 ± 0.48	1.89 (1.05, 3.39)
Rs2480258/Rs915906	*CYP2E1*	347			0.040
Other		331	1.03 ± 0.06	1
AA/GG		16	1.14 ± 0.33	1.53 (1.02, 2.28)
Rs2480258	*CYP2E1*	347			0.268
GG		203	1.02 ± 0.08	1
AG		119	1.05 ± 0.10	1.02 (0.86, 1.22)
AA		25	1.08 ± 0.25	1.38 (0.96, 1.98)
Rs915906	*CYP2E1*	347			0.265
AA		228	1.04 ± 0.08	1
AG		102	1.01 ± 0.10	1.07 (0.88, 1.29)
GG		17	1.09 ± 0.32	1.50 (0.99, 2.24)
Analyses carried out on 18 candidate genes with 89 SNPs available on the array. Only statistically significant relationships are reported. For the Rs2480258/Rs915906 SNPs, relationships estimated for the single SNPs are shown. ^***a***^Mean ± SE per 1,000 binucleated T lymphocytes. ^***b***^Mean ratio adjusted for country, maternal age, prepregnancy BMI, birth weight, sex, maternal ethnicity, gestational age, delivery, maternal smoking, and ETS. ^***c***^Log-likelihood ratio test, unadjusted for multiple comparisons.					

## Discussion

Here, we show that exposure biomarkers and T lymphocyte MN levels are measurable in cord blood, that large variations exist for these in the European newborn population, and also that some of the exposure biomarkers are associated with MN levels (as independent variables) and with gene polymorphisms (when the biomarkers are modeled as dependent variables). This suggests that the fetus may be exposed to carcinogenic chemicals *in utero* via the placenta, and that such exposures may be sufficient to exert early biological effects manifested as an increase in the frequency of MNBN, a marker that has been associated with cancer risk in adults (Bonassi et al. 2007). However, our findings should be interpreted with caution given that associations did not show evidence of consistent dose–response relations with increasing levels of exposure.

M_1_dG is the major DNA adduct arising from malondialdehyde, a genotoxic by-product of lipid peroxidation of polyunsaturated fatty acids with a high number of double bonds that also can be formed during food preparation (Jeong and Swenberg 2005). A significant overall association was detected between M_1_dG adduct levels and MNBN frequency, although the positive association was limited to the second and third quartiles, with the highest quartile of M_1_dG adducts being associated with the lowest MNBN frequency when compared with the lowest quartile. This association indicates recent exposure to malondialdehyde, because MNBN formation reflects recent genetic damage that results in micronuclei formation when cell replication is induced *in vitro*. No association was found between Hb adducts with MNMONO frequencies; however, fetal exposure to compounds detected by ERα CALUX and AR CALUX induced significant increases of MNMONO, possibly reflecting genetic damage accumulated during fetal development (Kirsch-Volders and Fenech 2001). The CALUX assays measure estrogenic, androgenic, or dioxin-like activities that could result from a variety of compounds or mixtures of compounds. Consequently, associations cannot be attributed to specific exposures. Infant acute leukemia is a frequent childhood cancer, and maternal exposure to hormones during pregnancy has been reported as a potential risk in disease occurrence (Pombo-de-Oliveira and Koifman 2006). A recent review (Holland et al. 2011) on MN in neonates and children concluded that exposure to environmental pollutants and radiation leads to increased MN; however, no information was provided on possible associations with other biomarkers of exposure and/or early effect, as presented in the present study.

The reduced number of samples available for the statistical analyses of the relationships between exposure biomarkers and MN levels is a limitation of the study and may have introduced false-negative findings. Conversely, some of the detected significant associations may have resulted from the multiple comparisons performed, increasing the chance of false-positive findings. In addition none of the observed associations followed a dose–response pattern.

We explored the expression of 36 genes by qRT-PCR as potential new biomarkers of toxic exposure. The expression of seven genes was negatively associated with MNBN (none with MNMONO), namely *SMC1A*, *LATS2*, *TRIM13*, *PDCD11*, *CD28*, *IL7R*, and *NIPBL*. The expression of these particular genes has previously been shown to be affected by one or more genotoxic carcinogens in experimental models (Mattingly et al. 2003). However, because detailed exposure data were absent, we could not further substantiate the involvement of specific chemicals. Using the dedicated TRANSFAC® software (BIOBASE Biological Databases, Beverly, MA, USA; http://www.biobase-international.com) for finding transcription factor expression in our transcriptomic data, we identified no transcription factor that could regulate all these genes. Given that MN are formed during metaphase/anaphase/telophase transition, it was of interest that most of the genes identified are involved in progression through the cell cycle, cell division, spindle formation, or DNA damage responses. *SMC1A* encodes a protein that is part of the cohesin protein complex and is involved in sister chromatid cohesion during the cell cycle (Bauerschmidt et al. 2011). The tumor suppressor gene *LATS2* encodes a protein that interacts with centrosome proteins and is required for correct spindle formation (Abe et al. 2006). *TRIM13* encodes a kinase involved in many different cellular processes including proliferation and apoptosis (Nakashima 2002). Furthermore, *CD28* and *PDCD11* are involved in apoptosis (Lacana and D’Adamio 1999; Walker et al. 1998). *NIPBL* is required for association of cohesin with chromosomes, for early processing of double-strand breaks and for the DNA damage checkpoint (Oka et al. 2011). For *IL7R*, the biological relevance for its association with MNBN remains unclear.

The expression of six of the seven genes associated with MNBN was also associated with the highest versus lowest level of one or more exposure biomarkers (Figure 1). *CD28*, *IL7R*, and *PDCD11A* were associated with the mutagenic DNA adduct M_1_dG. *CD28* and *PDCD11* are mainly involved in processes linked to genotoxic stress, such as apoptosis and cell cycle (Lacana and D’Adamio 1999; Walker et al. 1998). *LATS2* and *SMC1A* were associated with DR CALUX®, through which compounds that activate the transcription factor AhR, such as PCDDs (polychlorinated dibenzodioxins), PCDFs (polychlorinated dibenzofurans), dioxin-like PCBs (polychlorinated biphenyls), and PAHs (Pedersen et al. 2010) are measured; many of the latter are genotoxic. Activation of the AhR participates in pathways such as cell cycle regulation, apoptosis and immune responses (Marlowe and Puga 2005). Although *LATS2* and *SMC1A* are not known to be regulated by AhR, both genes are involved in certain subprocesses of the cell cycle. *NIPBL* was associated with AR CALUX®, which measures compounds with androgenic activity. Like AhR, AR is a transcription factor and regulates the expression of various genes involved in cell cycle control, apoptosis, cell growth, and differentiation (Heisler et al. 1997). Although *NIPBL* is not known to be regulated by AR, it is linked to genotoxic stress related processes and is involved in the cell cycle through its mediating function in sister chromatid cohesion (Watrin et al. 2006).

In summary, associations between gene expression profiles and MN induction reflect the origin of MN: Many of the genes are associated with chromosome breakage or loss, and particularly interference with spindle and chromatid segregation. Their associations with exposure biomarkers support their relevance in relation to genotoxic processes.

The analysis of genetic susceptibility was conducted using GWAS. A strong signal was observed on chromosome 11 for an interaction with AR CALUX® on MNBN frequency (see Supplemental Material, Table S2, Figure S4). The gene closest to this hotspot is *FOLH1* (folate hydrolase 1) and could thus be the genetic factor that affects this relationship. Several pseudogenes were closer, but were excluded because their function is unclear. *FOLH1*, also known as PMSA (prostate-specific membrane antigen), is overexpressed in prostate cancer and is negatively regulated by androgen (Ghosh et al. 2005). Furthermore, a polymorphism in *FOLH1* associated with lower levels of serum folate and hyperhomocysteinemia has been described (Devlin et al. 2000). Low folate is recognized as a risk factor for chromosome instability (Ames 2001) and MN induction (Fenech and Crott 2002). An interaction between androgen exposure and a polymorphism that modulates *FOLH1* expression might affect folate levels and thereby modify MNBN frequencies.

GWAS was carried out on 435 newborns with data available for both SNPs and micronuclei. The relatively small sample size is a limitation of the GWAS analysis and is likely to have introduced a risk of false-negative findings due to reduced statistical power to detect the studied associations. To reduce false-positive findings, we accounted for multiple comparisons in our primary GWAS analysis, although candidate gene analyses were not adjusted for multiple comparisons. We identified significant associations between *a priori*–selected SNPs in *EPHX1*, *EPHX2*, and *CYP2E1* and the frequency of MNBN lymphocytes (Table 4). These SNPs do not affect the protein code, but might be in linkage disequilibrium with causative variants. However, noncausal associations cannot be ruled out, and further clarification is required given inconsistent associations reported between these genes and MN in the literature (Dhillon et al. 2011).

In this study, samples from almost 1,200 newborns were collected. Because of limited sample volumes, the number of biomarker measurements varied from 1,151 for the AA–Hb adduct to 623 for MNBN, and 435 newborns had data available for both SNPs and micronuclei. For some analyses data were available for a limited number of observations: between 434 and 424 subjects for the associations between MNBN and candidate SNPs, and < 220 subjects for the interactions SNPs–exposure biomarkers on MNBN frequency. Although we were able to conduct association studies between individual exposure markers with MNBN, this seriously limited our ability to investigate the interaction between multiple exposure biomarkers and MNBN.

## Conclusions

We demonstrated that gene expression, lymphocyte MN levels, and a variety of biomarkers of environmental (geno)toxic exposure can be measured in newborn cord blood samples, and that there is interindividual variation in these markers in the European population. Associations of exposure biomarkers and genes (at the level of both gene expression and genotype) with MN frequencies may help generate new hypotheses about mechanisms of carcinogen-induced leukemia. The associations that we report must be interpreted with caution because we did not measure specific exposures, we did not observe monotonic dose–response relations, and we cannot rule out noncausal associations.

Nevertheless, our results suggest that internal exposure of the fetus to toxic chemicals occurs during apparently normal pregnancies, that such exposures may increase the frequency of MN formation [which, although of uncertain relevance in newborns (Holland et al. 2011) has been associated with cancer risk in adults (Bonassi et al. 2007)], and that some children may be more susceptible to genotoxic effects of *in utero* exposures than others.

Ultimately, information on the effects and sources of *in utero* genotoxic exposures could be used by regulators and industry to develop policy measures and strategies to reduce such exposures in order to improve children’s health and reduce the incidence of childhood cancer.

## Supplemental Material

(1.1 MB) PDF
