# Supplementary material for: Micronuclei in Cord Blood Lymphocytes and Associations with Biomarkers of Exposure to Carcinogens and Hormonally Active Factors, Gene Polymorphisms, and Gene Expression: The NewGeneris Cohort
Source: Environ Health Perspect. 2013 Nov 19;122(2):193–200. doi: 10.1289/ehp.1206324 (PMC3914866; doi:10.1289/ehp.1206324)
Supplement: (1.1 MB) PDF [file ehp.1206324.s001.508.pdf]

SUPPLEMENTAL MATERIAL

Micronuclei in Cord Blood Lymphocytes and Associations with Biomarkers of Exposure to Carcinogens and Hormonally Active Factors, Gene Polymorphisms, and Gene Expression: The NewGeneris Cohort

Domenico Franco Merlo, Silvia Agramunt, Lívia Anna, Harrie Besselink, Maria Botsivali, Nigel J. Brady, Marcello Ceppi, Leda Chatzi, Bowang Chen, Ilse Decordier, Peter B. Farmer, Sarah Fleming, Vicenzo Fontana, Asta Försti, Eleni Fthenou, Fabio Gallo, Panagiotis Georgiadis, Hans Gmuender, Roger W. Godschalk, Berit Granum, Laura J. Hardie, Kari Hemminki, Kevin Hochstenbach, Lisbeth E. Knudsen, Manolis Kogevinas, Katalin Kovács, Soterios A. Kyrtopoulos, Martinus Løvik, Jeanette K Nielsen, Unni Cecilie Nygaard, Marie Pedersen, Per Rydberg, Bernadette Schoket, Dan Segerbäck, Rajinder Singh, Jordi Sunyer, Margareta Törnqvist, Henk van Loveren, Frederik J. van Schooten, Kim Vande Loock, Hans von Stedingk, John Wright, Jos C. Kleinjans, Micheline Kirsch-Volders, Joost H.M. van Delft, and the NewGeneris Consortium*

* Other members of the NewGeneris consortium who participated in this study are: Ricardo Marcos, Diana Anderson, Elena Stagi, Viktória Lukács, Renee Mijal, and Ellen Nomark.

Table of contents

CALUX® methods for monitoring dioxin-like, androgen-like and estrogen-like agents 3
    DR-CALUX® Bioassay 3
ERα- and AR-CALUX® bioassays 4
DNA adducts 4
    Immunoslot blot analysis of M_1dG 4

Immuno-assays for O^6-MedG and PAH-DNA adducts 5
Standard O6-MedG or BPDE-modified DNA 5
DNA adduct analysis with the SCIA.. 6
Postlabelling analysis of bulky DNA adducts.. 6
Genome Wide Association Studies: population stratification 8
Supplemental Material, Table S1. Genes indicative for genotoxicity and/or immunotoxicity and the reference genes (ref) as selected for qRT-PCR analysis (Hochstenback et al. 2012). Reference genes are indicated as (ref) in the gene symbol column. 9
Supplemental Material, Table S2. GWAS analyses: SNPs identified as effect modifiers of the relationship between plasma AR-CALUX® and frequency of MNBN T-lymphocytes (see Figure S4).. 19
Supplemental Material, Figure S1. Multidimensional scaling approach addressing population stratification. Color coding in plot indicates country of birth and ethnicity defined as white Europeans (EU yes = Caucasians) or non white European (EU no = others). 20
Supplemental Material, Figure S2. Distribution of observed (green) and expected (black) p-values for tested SNP associations with MNBC using a negative binomial fixed effects multiple regression model adjusting for cohort, birth weight, child gender, maternal age, pre-pregnancy BMI, maternal ethnicity, gestational age, delivery type, maternal smoking and second hand smoke. Expected p-values computed under the null hypothesis (i.e., no association between SNPs and MNBC). 21
Supplemental Material, Figure S3. Manhattan plot for the associations between SNPs and the frequency of MNBN T-lymphocytes. The Y-axis shows -Log10 transformed P values, representing the level of the statistical associations. Higher and lower horizontal lines indicate p-values: <0.01 and <0.05 adjusted for multiple comparisons. 22
Supplemental Material, Figure S4. Manhattan Plot plasma AR-CALUX®-SNPs interaction and frequency of MNBN T-lymphocytes in newborns. The 4 SNPs reported in Table S2 are all on Chromosome 11 and cannot be identified due to the plot resolution. Higher and lower horizontal lines indicate p-values: <0.01 and <0.05 corrected for multiple comparisons. 23
References.. 24

CALUX® methods for monitoring dioxin-like, androgen-like and estrogen-like agents

DR-CALUX® Bioassay

Human blood was collected using heparinised tubes. Following blood collection, the tubes were centrifuged and the plasma fraction was collected and frozen at a minimum of -20°C. One to three ml of cord-blood plasma was used for extraction. Following addition of hexane:DEE (97:3 v/v), the plasma was vortexed for 2 min. The organic hexane layer was collected and evaporated until a volume of approximately 1 ml remained. Extractable fat and acid-labile matrix components were removed on a sulphuric acidic silica column topped with sodium sulphate. The final cleaned extract was evaporated under a gentle stream of nitrogen after which the remaining extract was re-dissolved in 8 µl of dimethyl sulfoxide (DMSO). The extractable fat content was determined gravimetrically.

The DR-CALUX reporter gene bioassay comprises a rat hepatoma cell line (H4IIE), stably transfected with an AhR-controlled luciferase reporter gene construct. Cells were cultured in α-MEM culture medium supplemented with 10% (v/v) FCS under standard conditions (37°C, 5% CO_2, 100% humidity). Cells were exposed in triplicate to extracts for 24 h in 96-well microtiter plates (0.8% DMSO). After incubation, the cells were lysed. A luciferine containing solution was added and the luciferase activity measured using a luminometer equipped with 2 dispensers (Berthold Centro XS3). Each 96-well microtiter plate contained a 2,3,7,8-TCDD calibration range. Total DR-CALUX TEQ content in the samples was determined by interpolation from the fitted 2,3,7,8-TCDD calibration curve.

ERα- and AR-CALUX® bioassays

For extraction, 0.5 ml of cord-blood plasma was used. Following addition of water, sodium phosphate buffer, K_2CO_3/$KHCO_3$ buffer and methyl tert-butyl ether (MTBE), the plasma was vortexed for 2 min. The organic MTBE layer was collected following centrifugation. MTBE was evaporated under a gentle stream of nitrogen after which the remaining extract was re-disolved in 40 µl of DMSO. The extractable fat content was determined gravimetrically.

Estrogenic and androgenic activity in human maternal and cord-blood plasma was determined using the ERα and AR-CALUX® Bioassays. ERα and AR-CALUX® cells were cultivated in DMEM medium supplemented with 7.5% (v/v) FCS under standard conditions (37°C, 5% CO_2 and 100% humidity). Prior to sub-culturing, cells were rinsed with PBS to remove debris. ERα and AR-CALUX® cells were seeded in 96-well microtiter plates and pre-incubated for 24 h. Following pre-incubation, cells were exposed to serial dilutions of the plasma extracts re-dissolved in DMSO. After 24 h of incubation the medium was removed and cells were lysed. Luminescence was detected in a luminometer equipped with 2 dispensers (Berthold Centro XS3) after addition of luciferin containing solution. All analyses were performed in triplicate. Each 96-well microtiter plate contained a calibration concentration series of 17ß-estradiol (ERα-CALUX®) or dihydrotestosterone (DHT) (AR-CALUX®). Total estrogenic and androgenic content was determined by interpolation from the fitted 17ß-estradiol or DHT calibration curve.

DNA adducts

Immunoslot blot analysis of M_1dG

Levels of M_1dG in human blood DNA samples were determined from a calibration line generated by the dilution (with control DNA) of standard calf thymus DNA containing known

amounts of M_1dG (ranging from 0 to 10 fmol M_1dG per µg DNA) pipetted onto the same immunoslot blot filter. The DNA standard was prepared by incubating calf thymus DNA with 1,1,3,3-tetramethoxypropane, and the level of malondialdehyde-guanine was determined by HPLC-fluorescence detection following acid hydrolysis. The Syngene ChemiGenius^2 image acquisition system (Synoptics Ltd., Cambridge, UK) was used to capture a chemiluminescent image of the filter. The adduct level in each sample was corrected for the amount of DNA bound to the filter as determined by propidium iodide staining.

Immuno-assays for O^6-MedG and PAH-DNA adducts

Levels of O^6-MedG and PAH-DNA adduct in blood DNA samples were determined from a calibration line generated, for each set of unknown samples analysed, by the dilution (with unmodified DNA) of standard DNA containing known amounts of adducts (ranging from 0 to 2 fmol adducts per reaction). In addition, samples of known adduct content were used in triplicate in each microtiter plate as quality control standards.

Standard O6-MedG or BPDE-modified DNA

Samples of DNA modified to approximately 1 O^6-MedG adduct/13,000 nucleotides were prepared with DNA from HeLa cells treated overnight at 37°C with N-methyl-N-nitrosourea, exhaustive purification and quantization after acid depurination (0.1 M HCl, 70°C, 30 min) and analysis of the supernatant containing bases on a Particil SCX HPLC column (Whatman, Maidstone, UK). Samples of DNA modified to approximately 1 adduct/6,000 nucleotides with BPDE were prepared by incubating HeLa cell DNA overnight in the dark at 37°C with BPDE dissolved in tetrahydrofuran, exhaustive purification and quantization using LC-MS/MS.

DNA adduct analysis with the SCIA

Standard adducted DNA, and the unknown DNA samples, were exhaustively digested overnight at 37^oC with MspI (New England Biolabs Inc., Ipswich, MA, USA) in 50 mM NaCl, 10 mM Tris-HCl, 10 mM $MgCl_2$, 1 mM dithiothreitol; pH 7.9. The DNA was subsequently denatured by heating to 95^oC for 10 min, snap-frozen in liquid nitrogen and then allowed to thaw on ice. Subsequently, an equal volume of anti-adduct–DNA rabbit antiserum (diluted in 2XPBST, 0.5% casein) was added to the DNA and the mixture was incubated at 37°C for 1.5 h in a shaking water-bath. One hundred µl of each DNA plus antiserum mixture was then transferred into triplicate wells of a microtiter plate pre-coated with goat anti-rabbit-IgG, and incubated for 1.5 h at room temperature, followed by five cycles of washing with PBST. Two sequential steps were then followed: 1) addition of 100 µl mouse anti-ssDNA antibodies, (Millipore Inc, Billerica, MA, USA) diluted 100-fold in PBST with0.25% casein and 2) addition of purified goat anti-mouse IgG antiserum conjugated with alkaline phosphatase, with minimum species cross-reactivity, (Biolegend, San Diego, CA, USA) diluted (1:500) in PBST containing 0.25% casein. CDP-Star containing Emerald II enhancer (Tropix, Bedford, MA, USA) was then added to the wells, the plates were incubated at room temperature for 30 min and luminescence was read using a Safire II Microplate Luminometer (TECAN, Männedorf, Germany) at 542 nm.

Postlabelling analysis of bulky DNA adducts

The levels of bulky DNA adducts were detected with the regular and modified ^{32}P-postlabelling procedure as detailed elsewhere (Kovács et al. 2011). DNA (4 µg) was digested with micrococcal nuclease and spleen phosphodiesterase to 3’-mononucleotides, followed by adduct enrichment with nuclease P1 and radiolabelling using carrier-free [γ-^{32}P]ATP and T4 polynucleotide kinase. DNA adducts were separated by multi-directional thin-layer

chromatography. The radioactive diagonal zone was identified and the associated radioactivity quantified by electronic autoradiograph. Background radioactivity in the blank area, corrected for the size of the adduct areas, was subtracted from the amount of radioactivity associated with the diagonal zone.

A standard protocol was applied by all three partners (Budapest, Hungary; Maastricht, the Netherlands; Stockholm, Sweden) involved in the analysis of samples. Inclusion of the same external BPDE-modified DNA standard (a DNA which had been reacted with benzo[a]pyrene-7,8-diol-9,10-epoxide and contained 111 adducts in 10^8 normal nucleotides (Phillips and Castegnaro 1999) allowed adjustment of data across sites and was a kind gift from Dr. Frederick F. Beland, NCTR, Arkansas, USA). All samples from Greece, Spain, Norway and the Danish samples collected in 2006-2007 were analysed in Budapest, Hungary (61% of the samples), the Danish samples from 2009 were analysed in Stockholm, Sweden (21%) and the samples from England in Maastricht, the Netherlands (18%). An inter-laboratory validation study was carried out where the same 23 DNA samples from the Bradford cohort were distributed among the three partners. The obtained data showed a very high repeatability between two of the three laboratories while the adduct levels measured in the third laboratory were consistently 3.7 times lower than the mean levels determined by the two other laboratories. Differences in DNA adduct determinations between laboratories are normally observed due to the complicated multi-step and sensitive procedures used for the detection of the adducts, and inter-laboratory studies are, therefore, necessary. A correction for the laboratory factor of 3.7 was applied to the samples analysed in Stockholm (Denmark 2009, 21% of the total). A sensitivity analysis that included or subsequently excluded these samples gave similar results and, therefore, all analyses were based on the total study population including the corrected data. The individual level of DNA adducts

was obtained as the average of at least two independent measurements. The detection limit of the assay was about 0.1-0.3 adducts per 10^8 unmodified nucleotides.

Genome Wide Association Studies: population stratification

Confounding by population stratification (i.e., the presence of genetic clusters), was assessed by using the multidimensional scaling approach (MDS) supported by the software Plink ver. 1.07, (http://pngu.mgh.harvard.edu/purcell/plink/) (Purcell et al. 2007). Individuals were plotted according to the first two estimated dimensions and coloured by country of birth and ethnicity with the aim of investigating whether genotype variations occurred between population subgroups (i.e., maternal ethnicity and country of birth, Figure S1). Visual inspection of the plot revealed the presence of genetic clusters related to the country of birth and ethnicity so these two covariates were included in all statistical models. To assess the extent of residual confounding due to population stratification, the Q-Q plot of the observed p-values for the SNPs-MNBC association, adjusted for potential confounders, against the p-values expected under the null hypothesis (i.e., no association between SNPs and MNBC) was done. As shown in Figure S2, no significant deviations between observed and expected values were detected indicating that genetic clustering was adequately controlled for in statistical analysis. The value of the genomic inflation factor (lambda) based on median and mean chi-squares statistics was 1.0 and 0.99, showing a very low level of inflation of the p-values.

Supplemental Material, Table S1. Genes indicative for genotoxicity and/or immunotoxicity and the reference genes (ref) as selected for qRT-PCR analysis (Hochstenback et al. 2012). Reference genes are indicated as (ref) in the gene symbol column.

GeneName	Gene symbol	TaqMan®Assay	Biological Process (www.geneontology.org) (Ashburner et al. 2000)
apoptotic chromatin condensation inducer 1	*ACIN1*	Hs00960114_m1	apoptosis (GO:0006915); positive regulation of monocyte differentiation (GO:0045657); apoptotic chromosome condensation (GO:0030263); erythrocyte differentiation (GO:0030218)
arachidonate 12-lipoxygenase	*ALOX12*	Hs00167524_m1	anti-apoptosis (GO:0006916); anti-apoptosis (GO:0006916); cellular component movement (GO:0006928); positive regulation of cell proliferation (GO:0008284); positive regulation of cell proliferation (GO:0008284); superoxide anion generation (GO:0042554); positive regulation of cell adhesion (GO:0045785); leukotriene biosynthetic process (GO:0019370); fatty acid oxidation (GO:0019395); positive regulation of cell growth (GO:0030307); oxygen and reactive oxygen species metabolic process (GO:0006800); oxidation reduction (GO:0055114)
anaphase promoting complex subunit 13	*ANAPC13*	Hs00612215_m1	mitosis (GO:0007067); cell division (GO:0051301); cell cycle (GO:0007049); protein K11-linked ubiquitination (GO:0070979); modification-dependent protein catabolic process (GO:0019941)
chromosome 10 open reading frame 46	*C10orf46*	Hs00403870_m1	ubiquitin-dependent protein catabolic process (GO:0006511)
chromosome 13 open reading frame 15	*C13orf15*	Hs00204129_m1	regulation of cyclin-dependent protein kinase activity (GO:0000079); cell cycle (GO:0007049)

GeneName	Gene symbol	TaqMan®Assay	Biological Process (www.geneontology.org) (Ashburner et al. 2000)
CD28 molecule	*CD28*	Hs00174796_m1	cell surface receptor linked signal transduction (GO:0007166); humoral immune response (GO:0006959); regulation of defense response to virus by virus (GO:0050690); cytokine biosynthetic process (GO:0042089); positive regulation of T cell proliferation (GO:0042102); positive regulation of anti-apoptosis (GO:0045768); positive regulation of mitosis (GO:0045840); regulatory T cell differentiation (GO:0045066); positive regulation of viral genome replication (GO:0045070); positive regulation of interleukin-2 biosynthetic process (GO:0045086); positive regulation of translation (GO:0045727); positive regulation of T cell proliferation (GO:0042102)
CD33 molecule	*CD33*	Hs00233544_m1	negative regulation of cell proliferation (GO:0008285); signal transduction (GO:0007165); cell adhesion (GO:0007155); cell-cell signaling (GO:0007267)
cyclin-dependent kinase 7 MO15 homolog, Xenopuslaevis, cdk-activating kinase	*CDK7*	Hs00387062_m1	nucleotide-excision repair, DNA damage removal (GO:0000718); DNA repair (GO:0006281); cell proliferation (GO:0008283); cell cycle (GO:0007049); response to DNA damage stimulus (GO:0006974); protein amino acid phosphorylation (GO:0006468); regulation of cyclin-dependent protein kinase activity (GO:0000079); cell division (GO:0051301); positive regulation of transcription from RNA polymerase II promoter (GO:0045944); regulation of transcription (GO:0045449); androgen receptor signaling pathway (GO:0030521); transcription initiation from RNA polymerase II promoter (GO:0006367); RNA elongation from RNA polymerase II promoter (GO:0006368)
CDC28 protein kinase regulatory subunit 1B	*CKS1B*	Hs02518862_g1	regulation of cyclin-dependent protein kinase activity (GO:0000079); cell cycle (GO:0007049); cell proliferation (GO:0008283); cell division (GO:0051301)

GeneName	Gene symbol	TaqMan®Assay	Biological Process (www.geneontology.org) (Ashburner et al. 2000)
collagen, type IV, alpha 3 Goodpasture antigen binding protein	*COL4A3BP*	Hs00178615_m1	cell morphogenesis (GO:0000902); mitochondrion morphogenesis (GO:0070584); heart morphogenesis (GO:0003007); in utero embryonic development (GO:0001701); lipid homeostasis (GO:0055088); response to endoplasmic reticulum stress (GO:0034976); cell proliferation (GO:0008283); signal transduction (GO:0007165); endoplasmic reticulum organization (GO:0007029); immune response (GO:0006955); muscle contraction (GO:0006936); lipid transport (GO:0006869); protein amino acid phosphorylation (GO:0006468); ceramide metabolic process (GO:0006672)
cathepsin G	*CTSG*	Hs00175195_m1	proteolysis (GO:0006508); immune response (GO:0006955); defense response to bacterium (GO:0042742)
Der1-like domain family, member 1	*DERL1*	Hs00251475_s1	retrograde protein transport, ER to cytosol (GO:0030970); response to unfolded protein (GO:0006986); ER-associated protein catabolic process (GO:0030433); intracellular transport of viral proteins in host cell (GO:0019060); protein transport (GO:0015031); endoplasmic reticulum unfolded protein response (GO:0030968); interspecies interaction between organisms (GO:0044419)
7-dehydrocholesterol reductase	*DHCR7*	Hs01023087_m1	multicellular organism growth (GO:0035264); lung development (GO:0030324); cell differentiation (GO:0030154); blood vessel development (GO:0001568); regulation of cholesterol biosynthetic process (GO:0045540); post-embryonic development (GO:0009791); regulation of cell proliferation (GO:0042127); oxidation reduction (GO:0055114)
enhancer of rudimentary homolog Drosophila	*ERH*	Hs00427977_m1	nucleobase, nucleoside, nucleotide and nucleic acid metabolic process (GO:0006139); cell cycle (GO:0007049); pyrimidine nucleoside metabolic process (GO:0006213)

GeneName	Gene symbol	TaqMan®Assay	Biological Process (www.geneontology.org) (Ashburner et al. 2000)
Fc fragment of IgE, high affinity I, receptor for alpha polypeptide	*FCER1A*	Hs00758600_m1	positive regulation of type I hypersensitivity (GO:0001812); positive regulation of calcium-mediated signaling (GO:0050850); serotonin secretion (GO:0001820); positive regulation of peptidyl-tyrosine phosphorylation (GO:0050731); positive regulation of granulocyte macrophage colony-stimulating factor biosynthetic process (GO:0045425); positive regulation of interleukin-3 biosynthetic process (GO:0045401); positive regulation of mast cell degranulation (GO:0043306); cell surface receptor linked signal transduction (GO:0007166); activation of JUN kinase activity (GO:0007257); leukotriene biosynthetic process (GO:0019370)
growth arrest and DNA-damage-inducible, beta	*GADD45B*	Hs00169587_m1	activation of MAPKKK activity (GO:0000185); activation of MAPKK activity (GO:0000186); apoptosis (GO:0006915); negative regulation of protein kinase activity (GO:0006469); regulation of cell cycle (GO:0051726); response to stress (GO:0006950); multicellular organismal development (GO:0007275); cell differentiation (GO:0030154)
interleukin 7 receptor	*IL7R*	Hs00902335_m1 (M)	regulation of DNA recombination (GO:0000018); homeostasis of number of cells (GO:0048872); negative regulation of T cell mediated cytotoxicity (GO:0001915); immunoglobulin production (GO:0002377); cell morphogenesis (GO:0000902); lymph node development (GO:0048535); B cell proliferation (GO:0042100); positive regulation of T cell differentiation in the thymus (GO:0033089); T cell differentiation (GO:0030217); cell growth (GO:0016049); cell surface receptor linked signal transduction (GO:0007166); immune response (GO:0006955)
linker for activation of T cells	*LAT*	Hs00175561_m1	intracellular signaling cascade (GO:0007242); mast cell degranulation (GO:0043303); calcium-mediated signaling (GO:0019722); Ras protein signal transduction (GO:0007265); regulation of T cell activation (GO:0050863); integrin-mediated signaling pathway (GO:0007229); immune response (GO:0006955)

GeneName	Gene symbol	TaqMan®Assay	Biological Process (www.geneontology.org) (Ashburner et al. 2000)
LATS, large tumor suppressor, homolog 2 Drosophila	*LATS2*	Hs00324396_m1	protein amino acid phosphorylation (GO:0006468); cell cycle (GO:0007049); mitosis (GO:0007067); protein kinase cascade (GO:0007243); hormone-mediated signaling pathway (GO:0009755); G1/S transition of mitotic cell cycle (GO:0000082); negative regulation of cyclin-dependent protein kinase activity (GO:0045736); cell division (GO:0051301)
mitogen-activated protein kinase kinase 6	*MAP2K6*	Hs00177150_m1	positive regulation of apoptosis (GO:0043065); response to drug (GO:0042493); activation of MAPK activity (GO:0000187); signal transduction (GO:0007165); cell cycle arrest (GO:0007050); DNA damage induced protein phosphorylation (GO:0006975); cardiac muscle contraction (GO:0060048)
mitogen-activated protein kinase kinase kinase 1	*MAP3K1*	Hs00394890_m1 (I)	protein polyubiquitination (GO:0000209); response to osmotic stress (GO:0006970); transforming growth factor beta receptor signaling pathway (GO:0007179); I-kappaB kinase/NF-kappaB cascade (GO:0007249); activation of JNKK activity (GO:0007256); activation of JUN kinase activity (GO:0007257); apoptotic mitochondrial changes (GO:0008637); positive regulation of gene-specific transcription from RNA polymerase II promoter (GO:0010552); peptidyl-serine phosphorylation (GO:0018105); regulation of cell migration (GO:0030334); positive regulation of actin filament polymerization (GO:0030838); negative regulation of actin filament bundle formation (GO:0032232); wound healing (GO:0042060); protein ubiquitination during ubiquitin-dependent protein catabolic process (GO:0042787); camera-type eye development (GO:0043010); positive regulation of apoptosis (GO:0043065); protein amino acid autophosphorylation (GO:0046777); positive regulation of viral transcription (GO:0050434); protein oligomerization (GO:0051259)

GeneName	Gene symbol	TaqMan®Assay	Biological Process (www.geneontology.org) (Ashburner et al. 2000)
Nibrin	*NBN*	Hs01039836_m1	DNA damage checkpoint (GO:0000077); neuromuscular process controlling balance (GO:0050885); telomere maintenance (GO:0000723); regulation of fibroblast proliferation (GO:0048145); negative regulation of neuron differentiation (GO:0045665); isotype switching (GO:0045190); response to drug (GO:0042493); positive regulation of kinase activity (GO:0033674); DNA duplex unwinding (GO:0032508); positive regulation of protein amino acid autophosphorylation (GO:0031954); G1/S transition checkpoint (GO:0031575); DNA damage response, signal transduction by p53 class mediator (GO:0030330); regulation of DNA replication initiation (GO:0030174); positive regulation of cell proliferation (GO:0008284); meiosis (GO:0007126); mitotic cell cycle G2/M transition DNA damage checkpoint (GO:0007095); cell cycle arrest (GO:0007050); in utero embryonic development (GO:0001701); blastocyst growth (GO:0001832); double-strand break repair (GO:0006302); response to DNA damage stimulus (GO:0006974); cell cycle (GO:0007049)
Nipped-B homolog Drosophila	*NIPBL*	Hs00209846_m1	cell cycle (GO:0007049); maintenance of mitotic sister chromatid cohesion (GO:0034088)

GeneName	Gene symbol	TaqMan®Assay	Biological Process (www.geneontology.org) (Ashburner et al. 2000)
nucleotide-binding oligomerization domain containing 2	*NOD2*	Hs01550762_g1	cytokine production during immune response (GO:0002367); protein oligomerization (GO:0051259); positive regulation of NF-kappaB transcription factor activity (GO:0051092); positive regulation of interleukin-1 beta secretion (GO:0050718); positive regulation of JNK cascade (GO:0046330); innate immune response (GO:0045087); positive regulation of I-kappaB kinase/NF-kappaB cascade (GO:0043123); regulation of apoptosis (GO:0042981); defense response to bacterium (GO:0042742); positive regulation of stress-activated MAPK cascade (GO:0032874); positive regulation of tumor necrosis factor production (GO:0032760); positive regulation of interleukin-17 production (GO:0032740); detection of muramyl dipeptide (GO:0032498); nucleotide-binding oligomerization domain containing 2 signaling pathway (GO:0070431); intracellular signaling cascade (GO:0007242); detection of bacterium (GO:0016045)
programmed cell death 11	*PDCD11*	Hs00958164_m1 (M)	mRNA processing (GO:0006397); rRNA processing (GO:0006364)
polymerase DNA-directed, delta 4	*POLD4*	Hs00221775_m1	positive regulation of endothelial cell proliferation (GO:0001938); nucleotide-excision repair, DNA gap filling (GO:0006297); DNA-dependent DNA replication (GO:0006261)
proteasome maturation protein	*POMP*	Hs00210902_m1	immune response (GO:0006955)

GeneName	Gene symbol	TaqMan®Assay	Biological Process (www.geneontology.org) (Ashburner et al. 2000)
protein kinase C, alpha	*PRKCA*	Hs00925195_m1	inactivation of MAPK activity (GO:0000188); positive regulation of smooth muscle cell proliferation (GO:0048661); regulation of receptor-mediated endocytosis (GO:0048259); response to antibiotic (GO:0046677); negative regulation of insulin receptor signaling pathway (GO:0046627); negative regulation of glucose import (GO:0046325); positive regulation of exocytosis (GO:0045921); negative regulation of heart contraction (GO:0045822); response to ethanol (GO:0045471); response to peptide hormone stimulus (GO:0043434); response to estradiol stimulus (GO:0032355); neutrophil chemotaxis (GO:0030593); central nervous system neuron axonogenesis (GO:0021955); peptidyl-threonine phosphorylation (GO:0018107); response to interleukin-1 (GO:0070555); positive regulation of synaptogenesis (GO:0051965); response to corticosterone stimulus (GO:0051412); induction of positive chemotaxis (GO:0050930); positive regulation of inflammatory response (GO:0050729); response to reactive oxygen species (GO:0000302); negative regulation of protein ami
RAD17 homolog S. pombe	*RAD17*	Hs00607830_m1	response to DNA damage stimulus (GO:0006974); DNA repair (GO:0006281); negative regulation of DNA replication (GO:0008156); DNA replication checkpoint (GO:0000076); mitotic cell cycle checkpoint (GO:0007093); cell cycle (GO:0007049); DNA damage checkpoint (GO:0000077); regulation of phosphorylation (GO:0042325)
structural maintenance of chromosomes 1A	*SMC1A*	Hs00196849_m1	chromosome organization (GO:0051276); DNA damage response, signal transduction (GO:0042770); response to radiation (GO:0009314); cell division (GO:0051301); DNA repair (GO:0006281); RNA splicing (GO:0008380); cell cycle (GO:0007049); mitotic spindle organization (GO:0007052); mitotic sister chromatid cohesion (GO:0007064); meiosis (GO:0007126); cell cycle checkpoint (GO:0000075); response to DNA damage stimulus (GO:0006974)
stromal antigen 3	*STAG3*	Hs00429370_m1	chromosome segregation (GO:0007059); synaptonemal complex assembly (GO:0007130); meiosis (GO:0007126); cell cycle (GO:0007049)

GeneName	Gene symbol	TaqMan®Assay	Biological Process (www.geneontology.org) (Ashburner et al. 2000)
toll-like receptor 4	*TLR4*	Hs00152939_m1	I-kappaB phosphorylation (GO:0007252); positive regulation of tumor necrosis factor production (GO:0032760); pathogen-associated molecular pattern dependent induction by symbiont of host innate immunity (GO:0052033); positive regulation of NF-kappaB transcription factor activity (GO:0051092); defense response to Gram-negative bacterium (GO:0050829); positive regulation of peptidyl-tyrosine phosphorylation (GO:0050731); positive regulation of inflammatory response (GO:0050729); positive regulation of JNK cascade (GO:0046330); negative regulation of osteoclast differentiation (GO:0045671); response to ethanol (GO:0045471); positive regulation of interleukin-8 biosynthetic process (GO:0045416); innate immune response (GO:0045087); positive regulation of interleukin-12 biosynthetic process (GO:0045084); positive regulation of nucleotide-binding oligomerization domain containing 1 signaling pathway (GO:0070430); positive regulation of nucleotide-binding oligomerization domain containing 2 signaling pathway (GO:0070434)
toll interacting protein	*TOLLIP*	Hs01553188_m1	immune response (GO:0006955); leukocyte activation (GO:0045321); phosphorylation (GO:0016310); cell-cell signaling (GO:0007267); intracellular signaling cascade (GO:0007242); inflammatory response (GO:0006954)
tripartite motif-containing 13	*TRIM13*	Hs00328634_s1	positive regulation of I-kappaB kinase/NF-kappaB cascade (GO:0043123); anatomical structure morphogenesis (GO:0009653)
TSC22 domain family, member 3	*TSC22D3*	Hs00608272_m1	regulation of transcription, DNA-dependent (GO:0006355)
tyrosine 3-monooxygenase/tryptophan 5-monooxygenase activation protein, zeta polypeptide	*YWHAZ*	Hs00852925_sH	response to drug (GO:0042493); protein targeting to mitochondrion (GO:0006626); histamine secretion by mast cell (GO:0002553); anti-apoptosis (GO:0006916); signal transduction (GO:0007165)

GeneName	Gene symbol	TaqMan®Assay	Biological Process (www.geneontology.org) (Ashburner et al. 2000)
ariadne homolog 2 (Drosophila)	*ARIH2 (ref)*	Hs00171838_m1	ubiquitin-dependent protein catabolic process (GO:0006511); multicellular organismal development (GO:0007275)
chromosome 3 open reading frame 10	*C3orf10 (ref)*	Hs00378446_m1	
eukaryotic translation initiation factor 3, subunit D	*EIF3D (ref)*	Hs00388727_m1	translational initiation (GO:0006413); translational initiation (GO:0006413)
proteasome (prosome, macropain) 26S subunit, ATPase, 1	*PSMC1 (ref)*	Hs02386942_g1	positive regulation of ubiquitin-protein ligase activity during mitotic cell cycle (GO:0051437); anaphase-promoting complex-dependent proteasomal ubiquitin-dependent protein catabolic process (GO:0031145); negative regulation of ubiquitin-protein ligase activity during mitotic cell cycle (GO:0051436); protein catabolic process (GO:0030163)
transmembrane protein 85	*TMEM85 (ref)*	Hs00212821_m1	apoptosis (GO:0006915)

Supplemental Material, Table S2. GWAS analyses: SNPs identified as effect modifiers of the relationship between plasma AR-CALUX® and frequency of MNBN T-lymphocytes (see Figure S4).

SNP	N	MNBN[a] Mean (SE)	AR-CALUX®[b] Mean (SE)	MR[c] (95%CI)	Nominal p-value[d]	Adjusted p-value [e]
rs11245676 / rs4568997[f]					$7.76e^{-8}$	0.020
AA	51	0.70 (0.11)	1.26 (0.08)	2.52 (1.69, 3.75)		
AG	110	0.84 (0.08)	1.32 (0.06)	1.25 (0.92, 1.69)		
GG	50	0.86 (0.12)	1.42 (0.07)	0.36 (0.21, 0.60)		
rs4881718					$1.33e^{-7}$	0.034
AA	50	0.71 (0.11)	1.27 (0.08)	2.49 (1.67, 3.72)		
AG	110	0.84 (0.08)	1.32 (0.06)	1.24 (0.92, 1.68)		
GG	51	0.84 (0.11)	1.40 (0.07)	0.36 (0.22, 0.62)		
rs7131537					7.90^{e-8}	0.020
AA	50	0.86 (0.12)	1.42 (0.07)	0.36 (0.21, 0.60)		
AC	109	0.84 (0.08)	1.32 (0.06)	1.24 (0.91, 1.68)		
CC	51	0.70 (0.11)	1.26 (0.08)	2.54 (1.69, 3.75)		

[a] per 1000 binucleated T-lymphocytes;

[b] AR-CALUX® expressed per 10^{-1} units of measurement (ng DHT AEQ/ml plasma*)*

[c] MR = Mean Ratio: change in the frequency of MNBN T-lymphocytes for a unit increase of plasma AR-CALUX® adjusted for cohort, birth weight, child gender, maternal age, pre-pregnancy BMI, maternal ethnicity, gestational age, delivery type, maternal smoking and second hand smoke.

[d] Nominal p-value of the LRT-test on the SNPs/exposure biomarker interaction term

[e] adjusted p-value for multiple comparisons

[f] Two SNPs with same subjects and slope

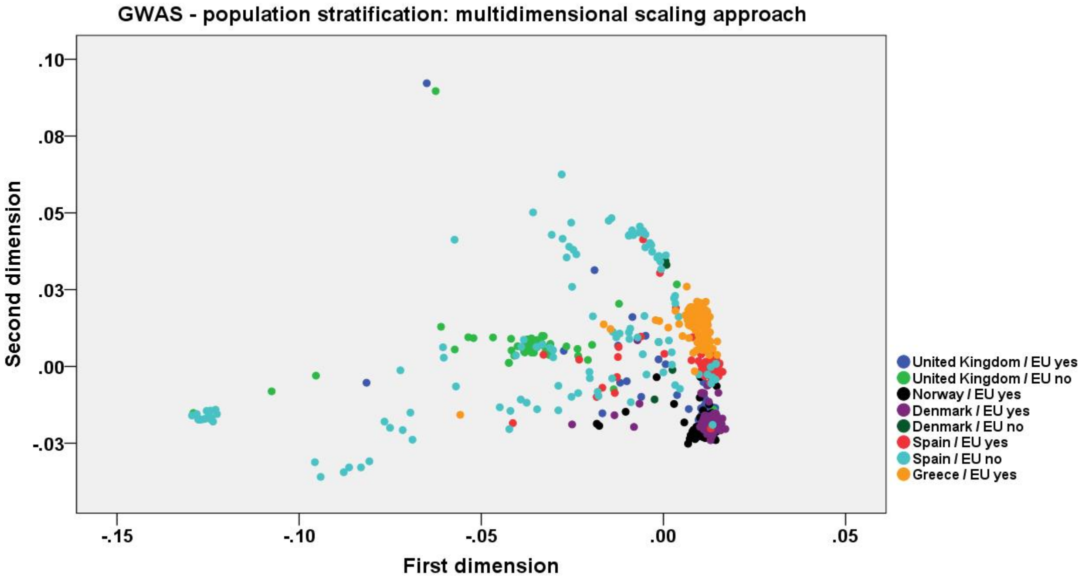

Supplemental Material, Figure S1. Multidimensional scaling approach addressing population stratification. Color coding in plot indicates country of birth and ethnicity defined as white Europeans (EU yes = Caucasians) or non white European (EU no = others).

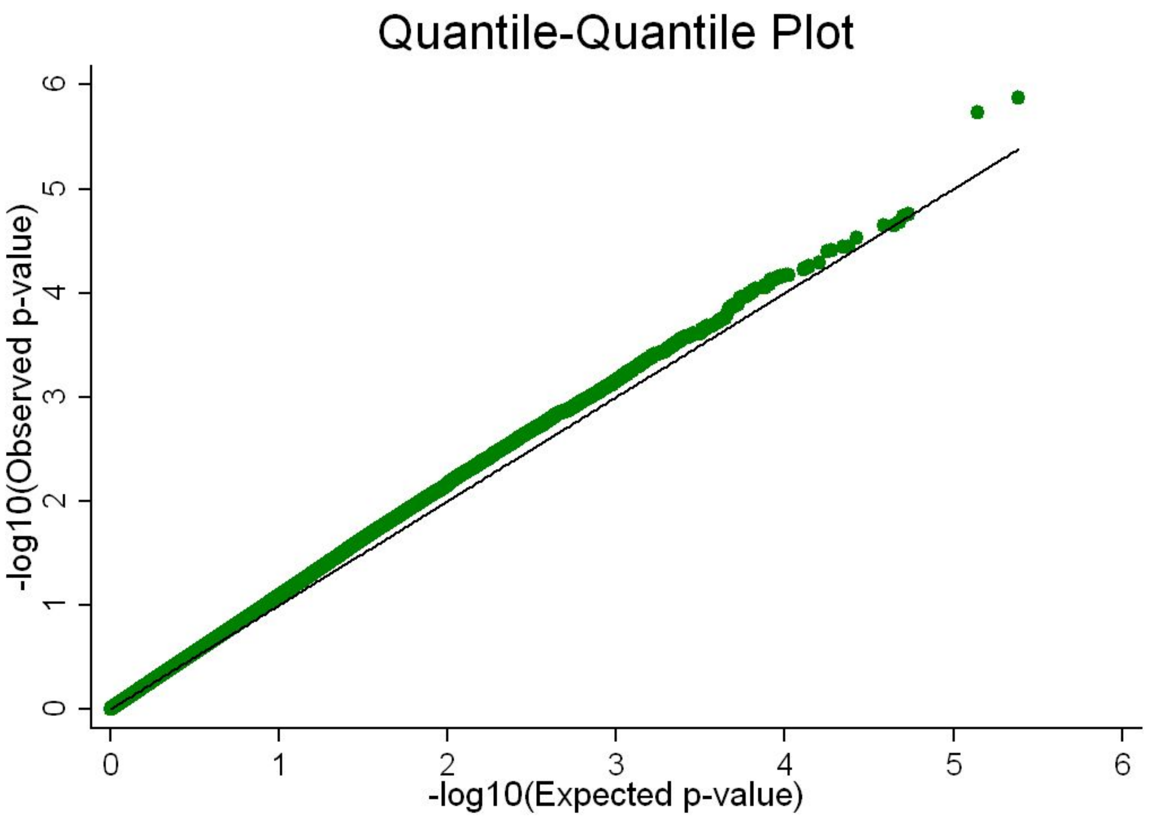

Supplemental Material, Figure S2. Distribution of observed (green) and expected (black) p-values for tested SNP associations with MNBC using a negative binomial fixed effects multiple regression model adjusting for cohort, birth weight, child gender, maternal age, pre-pregnancy BMI, maternal ethnicity, gestational age, delivery type, maternal smoking and second hand smoke. Expected p-values computed under the null hypothesis (i.e., no association between SNPs and MNBC).

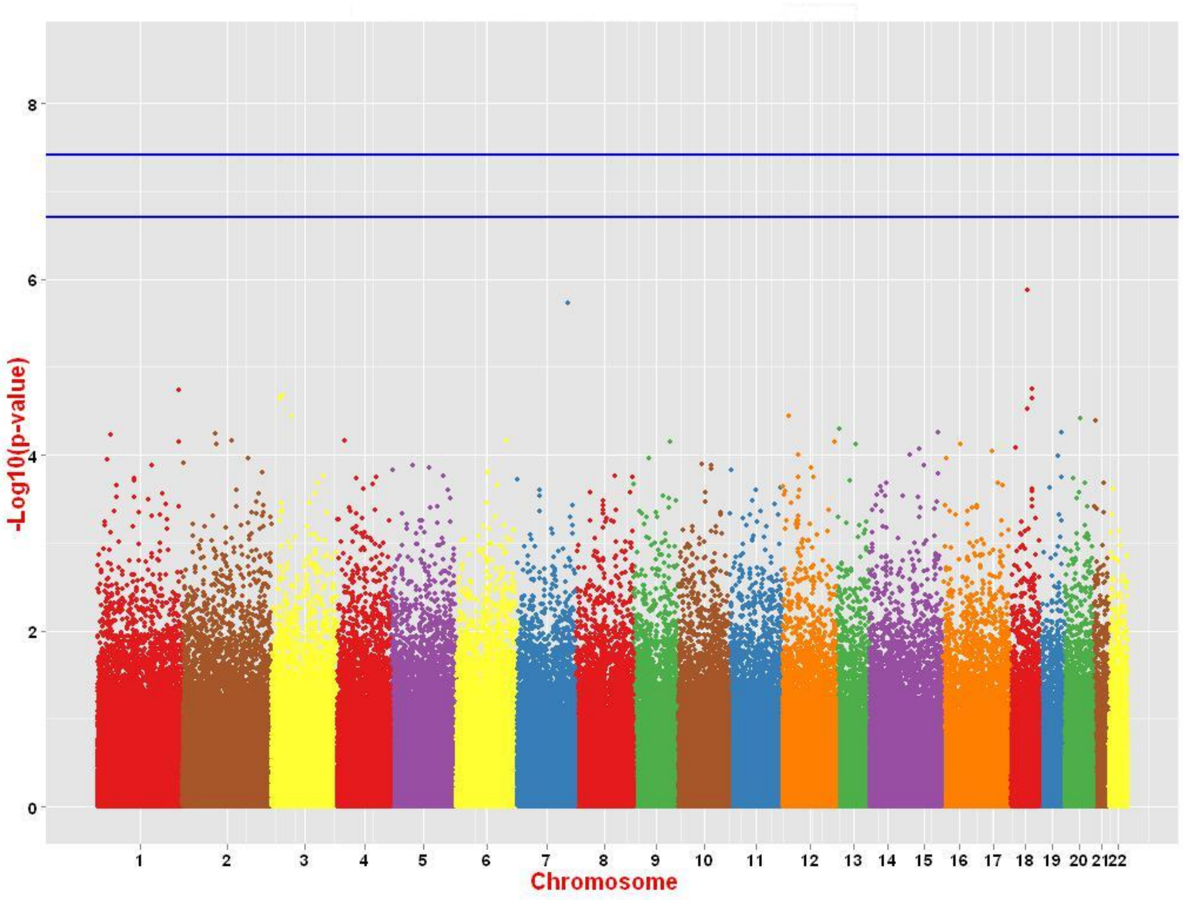

Supplemental Material, Figure S3. Manhattan plot for the associations between SNPs and the frequency of MNBN T-lymphocytes. The Y-axis shows -Log10 transformed P values, representing the level of the statistical associations. Higher and lower horizontal lines indicate p-values: <0.01 and <0.05 adjusted for multiple comparisons.

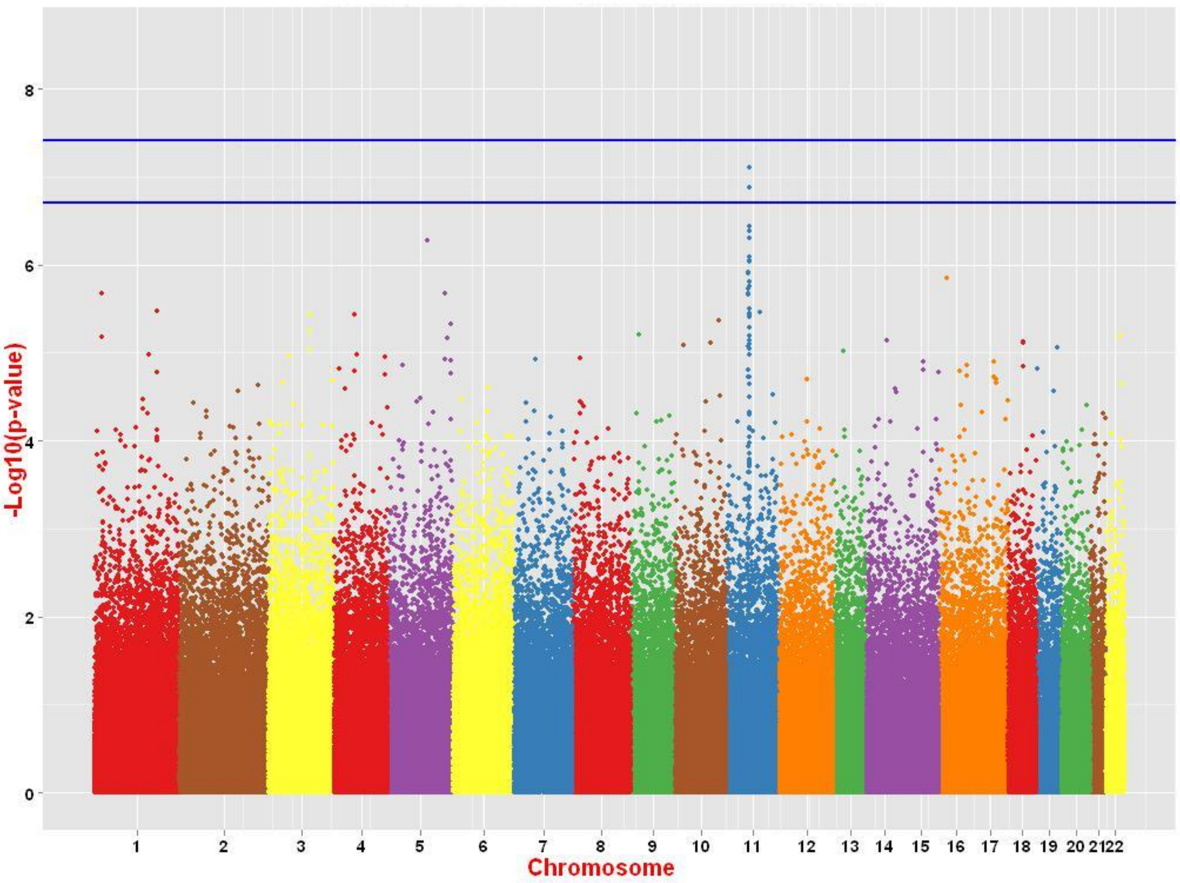

Supplemental Material, Figure S4. Manhattan Plot plasma AR-CALUX®-SNPs interaction and frequency of MNBN T-lymphocytes in newborns. The 4 SNPs reported in Table S2 are all on Chromosome 11 and cannot be identified due to the plot resolution. Higher and lower horizontal lines indicate p-values: <0.01 and <0.05 corrected for multiple comparisons.

References

Ashburner M, Ball CA, Blake JA, Botstein D, Butler H, Cherry JM et al. 2000. Gene Ontology: tool for the unification of biology. Nat Genet 25:25-29.

Hochstenbach K, van Leeuwen DM, Gmuender H, Gottschalk RW, Løvik M, Granum B et al. 2012. Global Gene Expression Analysis in Cord Blood Reveals Gender-Specific Differences in Response to Carcinogenic Exposure In Utero. Cancer Epid Biom Prev 10:1756-1767.

Kovács K, Anna L, Rudnai P, Schoket B. 2011. Recovery of bulky DNA adducts by the regular and a modified 32P-postlabelling assay; influence of the DNA-isolation method. Mutat Res 721:95-100

Phillips DH and Castegnaro M. 1999. Standardization and validation of DNA adduct postlabelling methods: report of intralaboratory trials and production of recommended protocols. Mutagenesis 14: 301-315.

Purcell S, Neale B, Todd-Brown K, Thomas L, Ferreira MA, Bender D et al. 2007. PLINK: a tool set for whole-genome association and population-based linkage analyses. Am J Hum Genet 81: 559-575.
